# Mass Spectrometry Profiling of Therapeutic Antibodies in Multiple Myeloma: *m*/*z* Features and Concordance with Immunofixation Electrophoresis †

**DOI:** 10.3390/biomedicines13122933

**Published:** 2025-11-28

**Authors:** Rosa Pello, María Ángeles Iglesias, Raúl Vidal, Raúl Mateos, Marta Outón, Cristina Agulló, Nerea Varo, Alberto Blanco-Sánchez, Nieves López-Muñoz, Álvaro García, Fátima Miras, Rodrigo Iñiguez, Daniel Gil-Alós, Rafael Alonso, Elena Ana López, Joaquín Martínez-López, María Teresa Cedena

**Affiliations:** 1 Department of Clinical Biochemistry and Laboratory Medicine, Hospital Universitario 12 de Octubre, 28041 Madrid, Spain; 2 Department of Clinical Biochemistry and Laboratory Medicine, Hospital Universitario de Salamanca, 37007 Salamanca, Spain; 3 Department of Clinical Biochemistry and Laboratory Medicine, Clínica Universidad de Navarra, 31008 Navarra, Spain; 4 Department of Hematology and Hemotherapy, Hospital Universitario 12 de Octubre, 28041 Madrid, Spain; 5 Instituto de Investigación Sanitaria Hospital 12 de Octubre (i+12), 28041 Madrid, Spain; 6 Faculty of Medicine, Complutense University of Madrid, 28040 Madrid, Spain; 7 H12O-CNIO Haematological Malignancies Clinical Research Unit, Spanish National Cancer Research Centre (CNIO), 28041 Madrid, Spain; 8 CIBERONC (CB16/12/00369), Instituto de Salud Carlos III (ISCIII), 28029 Madrid, Spain

**Keywords:** mass spectrometry, therapeutic antibodies, multiple myeloma, immunofixation electrophoresis (IFE), *m*/*z* profiling

## Abstract

**Background/Objectives:** Therapeutic monoclonal antibodies, including bispecifics (t-mAbs), can interfere with serum protein electrophoresis (SPEP) and immunofixation electrophoresis (IFE), mimicking residual M-protein. We evaluated a mass spectrometry (MS; EXENT^®^)-based workflow supported by an *m*/*z* reference library to discriminate drug from disease and assess concordance with IFE. **Methods:** Fifty-eight serum samples from 29 multiple myeloma patients were analyzed at baseline and after 3 months. Targeted enrichment of t-mAbs followed by MS enabled detection of peaks annotated through matching to a theoretical *m*/*z* panel and correlation with SPEP/IFE results. **Results:** Comparison of IFE versus MS showed 11/29 (38%) double positives, 15/29 (52%) double negatives, and 3/29 (10%) IFE−/MS+; no IFE+/MS− cases were observed. Using MS as a reference, IFE exhibited 78.6% sensitivity and 100% specificity. The *m*/*z* library enabled attribution of interference to linvoseltamab (*n* = 9), daratumumab (*n* = 6), and teclistamab (*n* = 3); in 16 patients treated with other bispecifics, no drug-related peaks were detected after 3 months. Longitudinal analysis discriminated therapeutic from endogenous immunoglobulins, identified baseline M-protein, and prevented false residual signals. **Conclusions:** MS (EXENT^®^)-based characterization of t-mAbs improves response monitoring accuracy in multiple myeloma and supports integration of MS into routine laboratory practice.

## 1. Introduction

Multiple myeloma (MM) and other plasma cell disorders are characterized by the clonal proliferation of plasma cells producing monoclonal immunoglobulins, commonly referred to as M-proteins. Traditionally, their detection and monitoring have relied on electrophoretic methods such as serum protein electrophoresis (SPEP) and immunofixation electrophoresis (IFE), complemented by serum free light-chain (FLC) analysis [1,2]. Although these techniques have been the cornerstone of MM diagnosis and follow-up during treatment monitoring, their sensitivity is increasingly insufficient in light of recent advances in targeted therapies and the growing emphasis on minimal residual disease (MRD) detection [3]. MRD assessment is essential for evaluating the depth of treatment response, predicting progression-free and overall survival, and guiding follow-up or therapeutic decisions [4].

In recent years, mass spectrometry (MS) has emerged as a valuable tool for the detection and monitoring of M-proteins [5]. Its high sensitivity and specificity enable non-invasive assessment of disease burden, potentially avoiding the need for invasive procedures such as bone marrow biopsy, representing a significant advancement in MM monitoring [6,7]. Moreover, MS can differentiate between endogenous M-proteins and therapeutic monoclonal antibodies (t-mAbs), such as daratumumab, which may interfere with the interpretation of conventional laboratory tests in patients receiving immunotherapy [8,9]. 

This study aims to characterize the specific mass-to-charge (*m*/*z*) ratios of the most commonly used t-mAbs in the treatment of MM and lymphomas. Accurate identification of these therapeutic antibodies by MS is essential to prevent misinterpretation, particularly to distinguish them from endogenous M-proteins during clinical assessment.

The primary objective of the present study is methodological rather than clinical. Specifically, this work aims to validate a mass spectrometry-based workflow, supported by a reference *m*/*z* library, for the analytical characterization and identification of therapeutic monoclonal and bispecific antibodies used in multiple myeloma and lymphomas. By establishing accurate *m*/*z* signatures for these antibodies, this study seeks to demonstrate the feasibility, analytical sensitivity, and precision of the EXENT^®^ system (The Binding Site, part of Thermo Fisher Scientific, Birmingham, UK) in distinguishing therapeutic antibodies from endogenous M-proteins.

In addition to the analytical characterization, a small exploratory cohort of 29 multiple myeloma patients treated with bispecific antibodies was included. Serum samples were collected at baseline (prior to treatment initiation) and three months after therapy initiation to illustrate the potential clinical applicability of the methodology in real patient samples, rather than to perform a clinical validation.

The resulting data provide valuable insights into the application of MS for t-mAb monitoring, enabling more accurate interpretation of laboratory findings and supporting informed clinical decision-making in patients with MM and lymphoma receiving novel immunotherapies [10]. Overall, this study is designed to provide proof-of-concept data demonstrating the robustness and analytical capability of MS for accurate t-mAb discrimination, while offering preliminary insight into its potential integration into clinical laboratory workflows.

## 2. Materials and Methods

### 2.1. Methods

The antibodies for analysis were selected based on their current clinical approval and inclusion in existing therapeutic protocols and ongoing clinical trials. Priority was given to those with the greatest impact on the monitoring of patients with monoclonal gammopathies, where accurate identification of the therapeutic t-mAb is crucial to avoid misclassification of relapse. 

Thus, *m*/*z* analysis was performed on selected monospecific and bispecific t-mAbs used in the treatment of MM—[11] daratumumab (DARA) (Janssen Biotech, Horsham, PA, USA), isatuximab (ISA) (Sanofi, Paris, France), elotuzumab (ELO) (Bristol Myers Squibb, New York, NY, USA), belantamab mafodotin (BELA) (GSK, London, UK), elranatamab (ELRA) (Pfizer, New York, NY, USA), teclistamab (TEC) (Janssen Biotech, Horsham, PA, USA), talquetamab (TAL) (Janssen Biotech, Horsham, PA, USA), linvoseltamab (LINVO) (Regeneron, Tarrytown, NY, USA), and etentamig (ETEN) (Regeneron, Tarrytown, NY, USA—and lymphomas—rituximab (RTX) (Genentech/Roche, South San Francisco, CA, USA), brentuximab vedotin (BV) (Seagen, Bothell, WA, USA), epcoritamab (EPCO) (Genmab/AbbVie, Copenhagen, Denmark; Chicago, IL, USA), odronextamab (ODRO) (Regeneron, Tarrytown, NY, USA), and mosunetuzumab (MOSU) (Genentech/Roche, South San Francisco, CA, USA) [12]. 

The antibodies were obtained from the surplus of the Pharmacy Service of our Institution.

For analytical characterization, t-mAbs were spiked into pooled serum samples from patients without detectable M-protein to a final concentration of 0.5 g/dL; this approach allowed us to confirm the presence of the antibody and to characterize it, except in cases where this was not feasible (because its initial concentration was less than 0.5 g/dL) such as ETE and MOSU, which were analyzed undiluted at their maximal available concentrations, 0.2 g/dL for ETE and 0.1 g/dL for MOSU [8]. 

Samples were processed in triplicate using MALDI-TOF mass spectrometry (EXENT^®^) with the EXENT^®^ system (The Binding Site, part of Thermo Fisher Scientific, Birminghan, UK). This allowed precise determination of the *m*/*z* values associated with each t-mAb. 

**Figure 1:**
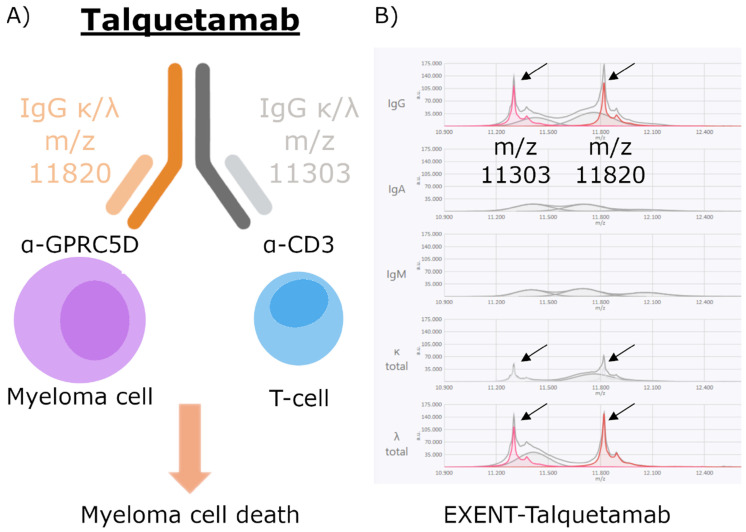
(**A**) Schematic representation of talquetamab (TAL), an IgGκλ bispecific t-mAb with dual specificity for GPRC5D and CD3, enabling T-cell-mediated myeloma cytotoxicity. (**B**) EXENT^®^ spectra showing the two-characteristic light-chain signals of the bispecific molecule. Peaks at *m*/*z* 11,303 and *m*/*z* 11,820 are detected across the IgG, κ, and λ panels because the immunocapture step isolates the intact bispecific antibody prior to reduction, resulting in both light chains appearing in each trace. Arrows highlight the characteristic mass spectral peaks at m/z 11,303 and m/z 11,820, corresponding to the κ and λ light chains of the bispecific antibody. These two peaks appear in the IgG, κ, and λ panels because the immunocapture step isolates the intact IgG bispecific molecule before reduction, resulting in both light chains being present in each trace. Arm-specific κ/λ assignment is inferred from mass signatures.

The EXENT^®^ technique, by using the Immunoglobulin GAM assay (The Binding Site, part of Thermo Fisher Scientific), employs polyclonal antibodies targeting IgG, IgA, IgM, and **intact light-chain** (**iLC**) kappa (**κ_iLC**) and lambda (**λ_iLC**), covalently bound to magnetic microparticles [13]. Upon immunoglobulin binding, disulfide bonds linking heavy and light chains are cleaved, releasing free light chains for analysis by matrix-assisted laser desorption/ionization time-of-flight mass spectrometry (MALDI-TOF-MS) [14]. This approach enables separation and identification of immunoglobulins based on their unique *m*/*z* signatures. The presence of a defined “peak” within an otherwise polyclonal spectrum is indicative of an M-protein. In the case of bispecific t-mAbs, two distinct peaks may appear, corresponding to the dual Fab (Fragment antigen-binding) regions of these therapeutic antibodies, as exemplified for TAL in Figure 1. This reflects the presence of both κ and λ light chains in TAL, a bispecific antibody containing both light-chain isotypes, which therefore produces signals in the κ and λ assays.

A tolerance of ±4 *m*/*z* units between the mean value of the triplicate measurements and the theoretical *m*/*z* was accepted, in accordance with the variability reported by the manufacturer [15].

Experimentally obtained *m*/*z* values were compared with theoretical values calculated from the amino acid sequences of **κ_iLC**, **λ_iLC**, or both, using the following bioinformatics tools: GenomeNet [16], ChEMBL [17], and ProtParam [18]. Amino acid compositions were retrieved from GenomeNet and ChEMBL, and theoretical molecular weights were calculated with ProtParam. The molecular weight was divided by two, assuming a predominant doubly charged ion species, to obtain the theoretical *m*/*z* of the t-mAbs. 

At the same time, SPEP was performed using the V8 Nexus capillary electrophoresis system (Helena Biosciences Europe, Gateshead, Tyne and Wear, UK; distributed in Spain by Menarini Diagnostics, S.A., Badalona, Spain), and IFE was carried out with the EasyFix^®^ Interlab G26 automated system (Interlab s.r.l., Rome, Italy; distributed in Spain by Sebia Hispania S.A), to confirm the presence of monoclonal components and determine the isotype [19]. 

### 2.2. Patient Samples

A total of 58 serum samples from 29 patients diagnosed with MM were analyzed. All patients were evaluated at relapse, before initiating a new line of therapy with t-mAb. Two time points were included: a baseline sample obtained at relapse (immediately before starting t-mAb) and a follow-up sample collected after three months of treatment. SPEP and IFE were performed at both time points as requested by the Hematology team for patient assessment. In addition, the SPEP and IFE performed at initial diagnosis were reviewed retrospectively and correlated with the EXENT^®^ findings. All samples were stored at −20 °C until analysis by EXENT^®^.

Patients were stratified according to MM isotype, as determined by IFE, and by the type of therapy received. The isotype distribution included IgGκ (*n* = 7), IgGλ (*n* = 5), IgAκ (*n* = 4), IgAλ (*n* = 4), Bence Jones κ (BJκ, *n* = 6), and Bence Jones λ (BJλ, *n* = 3). All patients were on treatment with bispecific monoclonal antibodies, either as monotherapy (ETEN, LINVO, TEC, and ELRA) or as part of combination regimens (TAL + DARA, TEC + TAL). The distribution of therapies by MM isotype is summarized in Table 1.

The three-month follow-up interval was selected to coincide with the first standardized clinical response assessment recommended by the International Myeloma Working Group (IMWG) [4]. This time point corresponds to approximately three treatment cycles and provides an early analytical snapshot of therapeutic antibody persistence and its differentiation from endogenous M-proteins, rather than a pharmacokinetic endpoint of complete drug clearance.

All patients provided written informed consent. The study was approved by the Institutional Research Ethics Committee in accordance with Spanish Biomedical Research Law 14/2007 and Royal Decree 1716/2011, and conducted in line with the principles of the Declaration of Helsinki.

## 3. Results

### 3.1. Monoclonal Antibody Characterization with EXENT^®^

Characterization data for t-mAbs used in the treatment of MM and lymphoma are summarized in Table 2. For most t-mAbs, the deviation between experimentally observed and theoretical *m*/*z* values remained within the accepted tolerance of ±4 *m*/*z* units, except for TAL, TEC, RTX, and EPCO, which showed a deviation of −10 *m*/*z* for one iLC, and BELA and BV additionally showed a signal at *m*/*z* 12,777 and 12,520.5, respectively. In general, the experimental *m*/*z* values showed excellent concordance with the theoretical masses predicted for t-mAbs with a difference of less than ± 4 units. 

To facilitate the interpretation of t-mAb detectability in clinical samples, Table 2 includes the published Cmax values for each therapeutic antibody [20].

**Figure 2:**
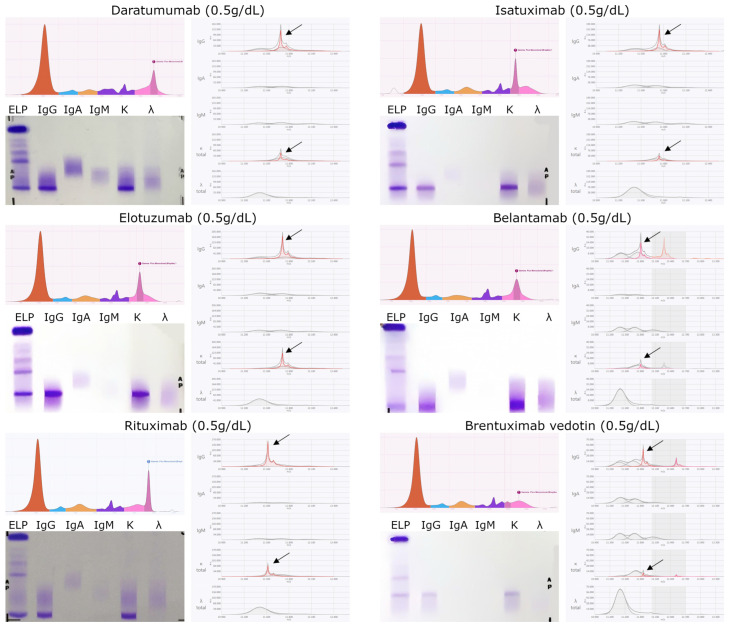
Representative data for each therapeutic monospecific t-mAb. On the left panels, SPEP and IFE results show migration patterns and confirm immunoglobulin isotype. On the right panels, EXENT^®^ spectra highlight the corresponding t-mAb peaks. Black arrows indicate therapeutic t-mAb signals, as referenced in Table 2.

**Figure 3:**
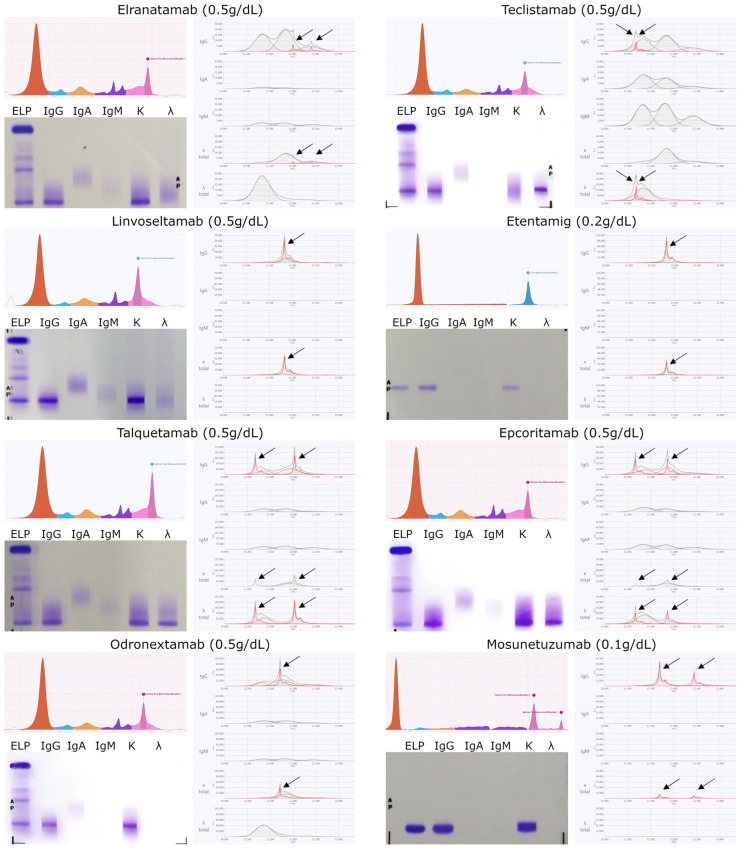
Representative data for each bispecific t-mAb. On the left panels, SPEP and IFE results display migration patterns and confirm immunoglobulin isotype. On the right panels, EXENT^®^ spectra highlight the peaks corresponding to t-mAbs used in MM and lymphoma. Black arrows indicate t-mAb signals, as referenced in Table 2.

The characterization of the t-mAbs was supplemented by SPEP and IFE, with results shown in Figure 2 and Figure 3. These analyses confirmed the isotype of each t-mAb and demonstrated that these drugs can produce peaks or bands of endogenous M-proteins.

In the case of ETE and MOSU, which were analyzed without a serum matrix, SPEP showed an early-migrating band that corresponds not to albumin but rather to the non-physiological migration of the purified therapeutic antibody. Purified proteins tend to migrate abnormally when run without serum proteins, producing a peak in the albumin region despite lacking immunological identity with albumin, which explains why this band is absent in immunofixation.

The reference preparations used for these analyses were enriched to concentrations higher than the physiological Cmax of the corresponding drugs (as detailed in Section 2.1), allowing the generation of clear, well-defined electrophoretic and EXENT^®^ spectral profiles. These standardized reference patterns facilitated the subsequent interpretation of t-mAb-related signals in clinical samples.

### 3.2. Analysis of Therapeutic Monoclonal Antibodies in Samples from Treated Patients

#### 3.2.1. Concordance Between EXENT^®^ and IFE

**Figure 4:**
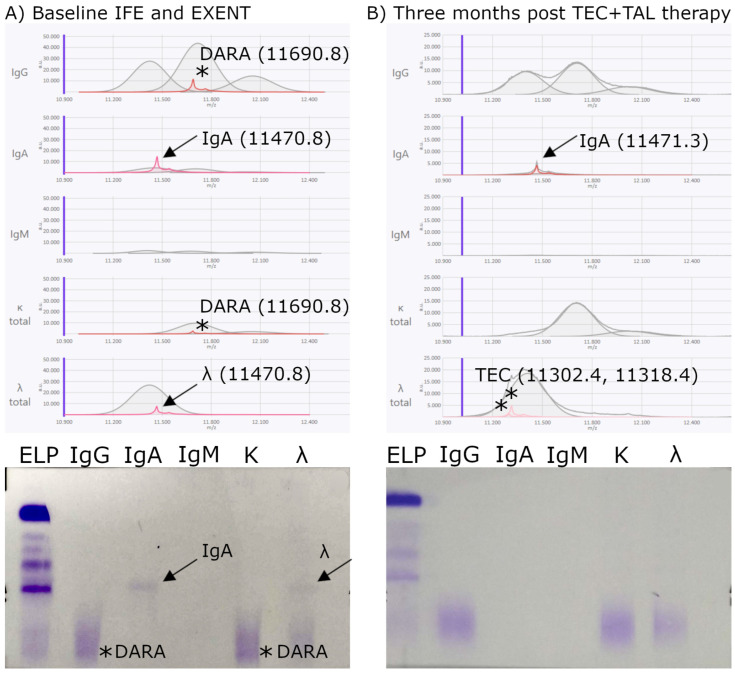
Discordant IFE/EXENT^®^ results. MM IgA λ with residual M-protein IgA peak after treatment. (**A**) At baseline, a faint IgAλ M-protein, close to the analytical detection limit, was detectable by IFE (bottom) and EXENT^®^ (top), with an *m*/*z* value of 11,470.8 and a concentration of 0.0148 g/dL. Additionally, a monoclonal IgGκ band was observed by IFE and confirmed by EXENT^®^ as a drug-related interference, with an *m*/*z* value of 11,690.8 and a concentration of 0.0149 g/dL, consistent with prior DARA therapy. (**B**) After 3 months of treatment with TEC + TAL, the IgAλ M-protein became undetectable by IFE; however, EXENT^®^ still revealed the presence of the patient’s endogenous M-protein (*m*/*z* 11,471.3). Two additional monoclonal λ components were detected by EXENT^®^, with *m*/*z* values of 11,302.4 and 11,318.4, corresponding to TEC. Arrows indicate the patient’s endogenous M-protein light-chain peaks detected by EXENT®, and the corresponding bands observed by IFE. Asterisks (*) mark t-mAb–related peaks (DARA at baseline and TEC-related λ peaks after 3 months).

**Figure 5:**
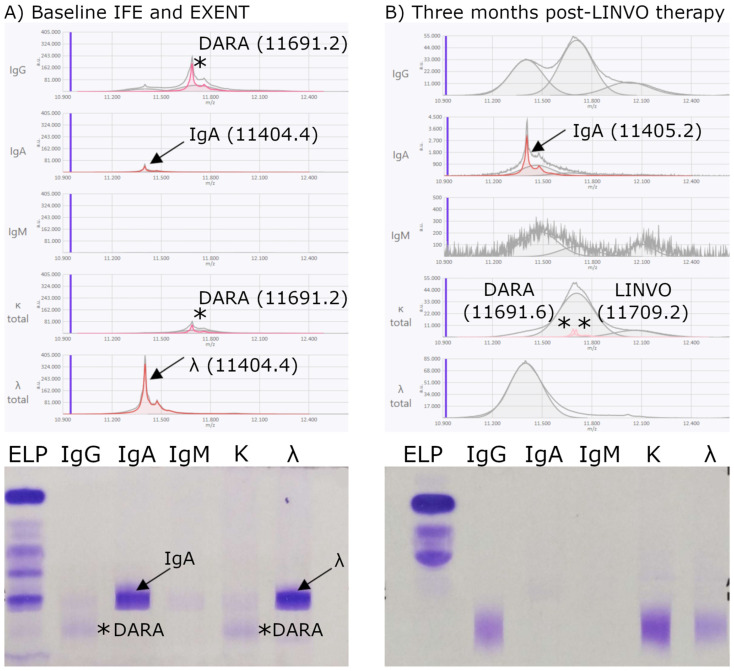
Discordant IFE/EXENT^®^ results. Residual IgA M-protein and LINVO interference in IgAλ MM treated with LINVO. (**A**) At baseline, an IgAλ M-protein was detectable by IFE (bottom) and EXENT^®^ (top), with an *m*/*z* value of 11,404.4 and a concentration of 0.565 g/dL. Additionally, a monoclonal IgGκ band was observed by IFE and confirmed by EXENT^®^ to be t-mAb interference, with an *m*/*z* value of 11,691.2 and a concentration of 0.0799 g/dL, consistent with prior DARA therapy. (**B**) After 3 months of LINVO, the IgAλ band became undetectable by IFE; EXENT^®^ continued to detect the patient’s endogenous M-protein (*m*/*z* 11,405.2) and **κ_iLC** corresponding to DARA (*m*/*z* 11,691.6) and LINVO (*m*/*z* 11,709.2). Arrows indicate the patient’s endogenous M-protein light-chain peaks detected by EXENT®, and the corresponding bands observed by IFE. Asterisks (*) mark t-mAb–related peaks, specifically DARA at baseline and both DARA- and LINVO-related peaks after 3 months.

**Figure 6:**
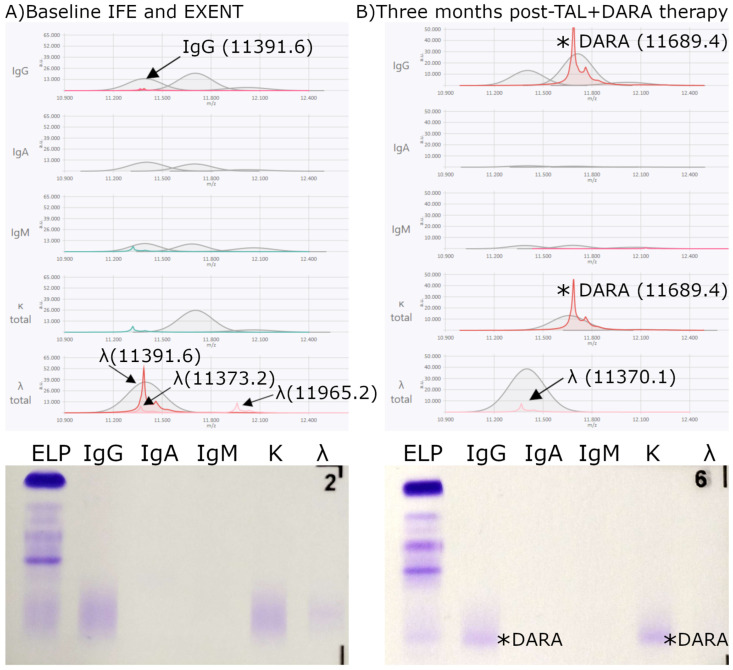
Discordant IFE/EXENT^®^ results. MM Bences Jones lambda only detected by EXENT^®^. (**A**) At baseline, IFE (bottom) lacked sufficient sensitivity to detect any M-proteins, whereas EXENT^®^ (top) identified two **λ_iLC** peaks (*m*/*z* 11,373.2 and 11,965.2) and one IgG λ with an *m*/*z* of 11,391.6 and a concentration of 0.0104 g/dL. (**B**) After three months of combination therapy with TAL + DARA, IFE remained negative, while EXENT^®^ revealed a persistent **λ_iLC** peak at *m*/*z* 11,370.1. A t-mAb–related IgGκ peak was observed by both techniques, consistent with DARA interference. Arrows indicate the endogenous λ_iLC and IgG λ peaks detected by EXENT® at baseline and the endogenous λ_iLC peak observed after three months of therapy. Asterisks (*) mark DARA-related peaks detected by both EXENT® and IFE.

At the baseline time point—defined as the relapse sample obtained immediately before initiating t-mAb therapy—the M-protein was detected in all 29 patients by both IFE and EXENT^®^. A second sample was collected after three months of treatment. These two time points were used to evaluate the concordance between IFE and EXENT^®^**.** Among the 29 patients, 11 (38%) patients were positive by both IFE and EXENT^®^, 15 (52%) were negative by both methods, and 3 (10%) were IFE-negative but EXENT^®^-positive (Figure 4, Figure 5 and Figure 6). No cases were observed in which IFE was positive and EXENT^®^ was negative.

Regarding **IFE-negative and EXENT^®^-positive patients**, as shown in Figure 4, for one of them, after 3 months of treatment with TEC + TAL, the IgAλ M-protein became undetectable by IFE; however, EXENT^®^ still revealed the presence of the patient’s endogenous M-protein (IgA), as the *m*/*z* value remained unchanged, albeit with a non-quantifiable concentration. Two additional λ_iLC peaks were detected by EXENT^®^, with *m*/*z* values of 11,302.4 and 11,318.4, corresponding to TEC. 

In the case of the second patient **IFE-negative/EXENT^®^-positive after 3 months of treatment with LINVO**, as shown in Figure 5, the IgAλ band became undetectable by IFE, although residual DARA may still be detectable, consistent with prior DARA therapy; however, EXENT^®^ continued to detect the patient’s endogenous M-proteins, as the *m*/*z* value remained unchanged (11,404.4), albeit at a concentration below the quantification limit. Additionally, κ_iLC peaks corresponding to both DARA (*m*/*z* 11,691.6) and LINVO (*m*/*z* 11,709.2) were also detected by EXENT^®^. 

The third discordant patient was a **Bences Jones lambda patient**, where, at baseline (Figure 6, left panel), IFE was not sensitive enough to detect any M-proteins, whereas EXENT^®^ identified two λ_iLC peaks (*m*/*z* 11,373.2 and 11,965.2) and one IgGλ with an *m*/*z* of 11,391.6 and a concentration of 0.0104 g/dL. After three months of combination therapy with TAL + DARA (Figure 6B), IFE remained negative, while EXENT^®^ continued to detect a persistent λ_iLC peak at *m*/*z* 11,370.1. An IgGκ M-protein was observed by both techniques, consistent with Dara-related interference.

#### 3.2.2. Analysis of Interferences from Monoclonal and Bispecific Antibodies

##### Patients with Detectable Therapeutic Antibody Interference

Interference attributable to LINVO was identified in nine patients: three with the IgAλ isotype, three with BJκ, two with IgGλ, and one with IgGκ. After three months of treatment with LINVO, EXENT^®^ consistently detected a peak corresponding to the IgGκ or κ_iLC isotype at *m*/*z* 11,708 ± 4 (Figure 5), matching the expected *m*/*z* for LINVO, as listed in Table 2.

Interference attributable to **DARA** was observed in six patients, all of whom were receiving combination therapy with TAL, a bispecific antibody not detected by EXENT^®^ in any of the cases studied. Among these patients, four had IgAκ MM, one had BJ κ MM, and one had BJ λ MM. Additionally, DARA was detected in one patient both at baseline and after LINVO treatment (Figure 5). This patient had previously received DARA.

Interference attributable to **TEC** was identified in three patients: one with IgAλ MM (Figure 4) and two with IgGκ MM. In all cases, EXENT^®^ detected λ_iLC peaks at *m*/*z* 11,302 ± 4 and *m*/*z* 11,317 ± 4, consistent with the expected *m*/*z* values for TEC, as reported in Table 2.

##### Patients Without Detectable Therapeutic Antibody Interference

A total of 16 patients received other bispecific antibodies including **TAL** (Figure 4 and Figure 6), **ELRA**, and **ETEN**. In all these cases, no peaks corresponding to the t-mAbs were detected by EXENT^®^ after three months of treatment. The only exception was a patient treated with ETEN, in whom an IgGκ peak at *m*/*z* 11691 was detected both at baseline and post-treatment. Since this *m*/*z* value corresponds to both ETEN and DARA, and the peak was already present prior to ETEN administration, it is likely that the signal reflects residual DARA from prior treatment line.

## 4. Discussion

The precise characterization of the *m*/*z* values of t-mAbs used in the treatment of MM and lymphomas represents a significant advancement for clinical laboratories, as it enables accurate discrimination of these t-mAbs from endogenous M-proteins in patients receiving immunotherapy. In line with this, the International Myeloma Working Group (IMWG) has recently recommended the use of MS approaches, such as iLC MALDI-TOF, over traditional IFE, owing to their superior sensitivity and their ability to differentiate t-mAbs from disease-related immunoglobulins [7,21]. This is particularly relevant in IgG-type MM patients treated with DARA, ISA, and ELO or bispecific antibodies like TAL or TEC, where overlapping bands on SPEP or IFE may otherwise lead to misclassification of residual disease or relapse. MS-based platforms such as EXENT^®^ overcome these limitations by resolving drug-specific *m*/*z* signatures that gel-based techniques cannot reliably distinguish [22].

IFE interference varies according to the underlying myeloma isotype and is predominantly observed in IgG myelomas, as most therapeutic antibodies used in MM are IgG-based and co-migrate within the IgG region. In contrast, IgA, IgD, and light-chain-only myelomas typically exhibit minimal or no interference, owing to their distinct electrophoretic behavior or the absence of a heavy chain. Consequently, our dataset does not demonstrate complete resolution of IFE interference across all isotypes; instead, it highlights the superior analytical sensitivity and discriminative capability of mass spectrometry compared with IFE.

Supporting this, the data presented in Table 2 demonstrate a high concordance between the experimentally observed and theoretical *m*/*z* values of the analyzed t-mAbs, with only minor deviations in some antibodies, such as TAL, TEC, EPCO, BELA, and BV. Specifically, TAL, TEC, RTX, and EPCO exhibited a deviation of −10 *m*/*z* for one of their iLCs, likely attributable to post-translational modifications, whereas BELA and BV—both conjugated antibodies—were consistent with signals detected at *m*/*z* 12,777 and 12,520.5 units, respectively (Figure 2). These findings validate the robustness of EXENT^®^ for precise drug-specific *m*/*z* identification and, to our knowledge, represent the first application of this platform in lymphoid malignancies.

The characterization of the different *m*/*z* values of t-mAbs (Table 2) enabled the identification of peaks observed by EXENT^®^ in samples processed three months after treatment, as shown in Figure 4, Figure 5 and Figure 6, where the *m*/*z* values correlated with the t-mAbs TEC, LINVO, and DARA, respectively. Overall, the detectability of TEC, LINVO, and daratumumab in our cohort is fully consistent with their higher clinical Cmax values. Notably, the *m*/*z* values obtained for TEC are in agreement with the independent data reported by Nevejan at EuroMedLab 2025 [23], further supporting the accuracy of our mass assignments. In particular, interference with DARA was detected, as illustrated in Figure 5, supporting the hypothesis of either drug carryover or prolonged persistence. Consistent with our findings, several studies have reported the persistence of therapeutic antibodies after treatment; for example, MASS-FIX studies demonstrated that DARA can remain detectable for months following drug discontinuation [24].

This persistence of DARA in serum has been the main rationale for the development of reflex assays such as Hydrashift or DIRA Test, specifically designed to resolve DARA interference in IFE, since its bands may be mistaken for endogenous monoclonal proteins. More recently, the DIRA assay has also been adapted to address interference from ISA, extending its utility beyond DARA. Nevertheless, the applicability of these tests remains limited to this particular subset of therapeutic antibodies (24). In the present series, no evidence of other t-mAbs, such as **TAL** (Figure 4 and Figure 6), **ELRA**, or **ETEN**, was detected in the analyzed samples. This lack of detection can be fully explained by their pharmacokinetic properties and analytical behavior. TAL has a very low clinical Cmax (1.6–3.8 µg/mL), well below the practical detection limit of EXENT^®^ (~15 µg/mL), which is consistent with the absence of TAL in patient samples and with the *m*/*z* values independently reported in the EuroMedLab 2025 poster by Nevejan et al. [23]. In contrast, ELRA and ETEN reach higher Cmax values, but their detection in routine samples is limited by several factors: sampling at month 3 rather than at peak concentration, marked interindividual variability in drug exposure, common dose delays or reductions, and the intrinsically weaker iLC peak intensity often observed for bispecific antibodies. Bispecifics can exhibit reduced analytical recovery due to their dual-light-chain composition and structural heterogeneity, which may compromise reduction efficiency and signal yield [7]. Although ELRA and ETEN were clearly identifiable in vitro when analyzed at higher concentrations, their detection in patients may require sampling closer to Cmax or evaluation in larger cohorts.

As a limitation, it should be considered that t-mAbs may share the same *m*/*z* or one very close to that of the patient’s M-protein, which may compromise their discrimination by mass spectrometry. This overlap has been described for DARA by Moore et al., 2019 [10].

Furthermore, longitudinal comparison of patient samples before and after therapy permitted not only the discrimination of therapeutic from endogenous immunoglobulins but also typification of the original M-protein peak (Figure 4, Figure 5 and Figure 6). This capability is crucial to distinguish residual neoplastic M-protein from treatment-derived bands, thereby enhancing clinical decision-making accuracy.

However, baseline studies are not always available, particularly in patients referred from other centers, and the lack of information on the administered therapy may lead to misinterpretation. As shown in Figure 2 and Figure 3, t-mAbs can be mistakenly identified as endogenous M-proteins, either as a band on IFE or as a peak by MS, and thus incorrectly reported as positive disease if their therapeutic origin is not recognized. In these scenarios, the laboratory report should include an interpretive comment indicating the potential superposition, especially when treatment history is incomplete or unavailable [25]. This risk is especially relevant in patients treated with IgG-type t-mAbs who also present an IgG-type M-protein, where IFE may lead to misinterpretation. In contrast, EXENT^®^, through MS and the resolution of the specific *m*/*z* of the t-mAb, enables accurate characterization, thereby supporting correct interpretation and ultimately improving diagnostic accuracy.

IFE demonstrated a sensitivity of 78.6% and a specificity of 100% when compared to EXENT^®^. Specificity was calculated as a comparative methodological parameter between techniques (using IFE as the reference), considering as false positives those cases that were negative by IFE but positive by EXENT. This measure does not reflect clinical specificity. These results highlight the superior analytical sensitivity of EXENT^®^, which was able to detect monoclonal peaks missed by conventional IFE (Figure 4, Figure 5 and Figure 6), particularly in patients with low disease burden or receiving monoclonal antibody therapy. According to the current IMWG response criteria, such a patient would be classified as being in complete remission. However, in light of the recent results demonstrating the higher sensitivity and specificity of mass spectrometry—comparable to other high-sensitivity techniques such as next-generation flow or next-generation sequencing [26,27]—a new response category reflecting deep remission assessed by mass spectrometry should be considered for inclusion in the next revision of the multiple myeloma response criteria.

Our findings are consistent with previous studies showing that MS outperforms IFE in sensitivity and specificity, allows detection of lower levels of MRD, and reliably distinguishes therapeutic antibodies from endogenous M-protein [28]. Moreover, clinical data have demonstrated that patients in whom MS fails to detect M-proteins exhibit improved clinical outcomes, suggesting that MS negativity is a more accurate predictor of prognosis than IFE, and reinforcing its role as a tool for MRD monitoring and as a non-invasive alternative to bone marrow biopsy [29]. In this context, IMWG has emphasized the potential of MS to progressively replace conventional SPEP and IFE in clinical monitoring, while underscoring the need for standardization, multicenter validation, and gradual integration into diagnostic algorithms for multiple myeloma [7].

From a laboratory perspective, EXENT^®^ demonstrated high precision and reliability in differentiating t-mAbs from endogenous M-proteins. The strong concordance between experimental and theoretical *m*/*z* values validates EXENT^®^ as a robust tool for accurate antibody identification. This capability is crucial to avoid misinterpretation of laboratory results, particularly in patients receiving immunotherapy, and its superior analytical sensitivity was evident in detecting monoclonal peaks missed by conventional techniques such as SPEP and IFE, especially in cases with low disease burden.

In this context, establishing the **baseline *m*/*z* of the M-protein at diagnosis** is essential for accurate typing and for the reliable interpretation of peaks during and after treatment. Likewise, knowledge of the **therapeutic regimen administered** is equally critical, since therapeutic monoclonal antibodies can generate peaks that overlap with or mimic endogenous M-proteins, thus complicating interpretation. 

Clinically, the integration of EXENT^®^ into routine practice enhances treatment monitoring by enabling accurate distinction between t-mAbs and residual neoplastic M-proteins. This provides a sensitive, specific, and non-invasive method for detecting residual disease in peripheral blood, improving patient comfort and compliance. Moreover, the ability of EXENT^®^ to detect t-mAb interference and differentiate it from endogenous M-proteins ensures accurate assessment of treatment efficacy and supports more precise therapeutic decisions, ultimately contributing to better patient outcomes.

Limitations of our study include the relatively small patient cohort and limited evaluation of newer t-mAbs. Future studies with larger cohorts and extended follow-up are necessary to validate these findings and promote EXENT^®^ or similar mass spectrometry platforms as standard tools in the immunotherapy era.

## Figures and Tables

**Table 1:** Distribution of bispecific t-mAb therapies by MM isotype.

		Bispecific t-mAb Therapies					
MM Isotype	Total	ETEN	LINVO	TEC	TAL + DARA	TEC + TAL	ELRA
IgGκ	7	4	1	2			
IgGλ	5	1	3	1			
IgAκ	4				4		
IgAλ	4		3			1	
BJ κ	6	1	3		1		1
BJ λ	3	1			1		1

This table summarizes the number of patients per isotype and the corresponding treatment received. MM: multiple myeloma; Ab: antibody; ETEN: etentamig; LINVO: linvoseltamab; TEC: teclistamab; TAL: talquetamab; DARA: daratumumab; ELRA: elranatamab.

**Table 2:** Theoretical and experimentally measured *m*/*z* values, immunoglobulin isotypes, antigen targets of t-mAb used in the treatment of MM and lymphomas, and maximum t-mAb concentration (Cmax).

		Monoclonal Antibody	Theoretical *m*/*z*	Measured *m*/*z*	Isotype	Target	Cmax (µg/mL)
Monospecific	MM	Daratumumab	11,692.0	11,691.1 ± 0.1	IgGK	Anti CD38	132–780 (SC)/256–688 (IV)
		Isatuximab	11,745.1	11,744.5 ± 0.4	IgGK	Anti CD38	351
		Elotuzumab	11,713.5	11,713.4 ± 0.3	IgGK	Anti SLAMF7	217–226
		Belantamab	11,816.2	11,815.4 ± *	IgGK	Anti BCMA	43
	Lymph	Rituximab	11,528.3	11,519.7 ± 0.4	IgGK	Anti CD20	157–404
		Brentuximab vedotin	11,864.1	11,864.2 ± 0.5	IgGK	Anti CD30	31.98
Bispecific	MM	Elranatamab	11,812.2	11,811.5 ± 0.3	IgGK	Anti BCMA	20.1–33.6
			12,050.4	12,049.6 ± 0.2	IgGK	Anti CD3	
		Teclistamab	11,312.2	11,302.9 ± 0.4	IgGL	Anti CD3	
			11,318.0	11,317.6 ± 0.6	IgGL	Anti BCMA	23.8–25.3
		Linvoseltamab	11,709.0	11,708.4 ± 0.8	IgGK	Anti CD3 BCMA	64.8–124
		Etentamig	11,697.0	11,695.6 ± 0.1	IgGK	Anti CD3 BCMA	100–200
		Talquetamab	11,312.2	11,303.3 ± 0.1	IgGKL	Anti CD3	1.6–3.8
			11,820.6	11,820.4 ± 0.1	IgGKL	Anti GPRC5D	
	Lymph	Epcoritamab	11,311.7	11,301.7 ± 0.5	IgGKL	Anti CD3	4.76–11.1
			11,721.0	11,719.3 ± 0.5	IgGKL	Anti CD20	
		Odronextamab	11,629.0	11,628.1 ± 0.2	IgGK	Anti CD3 Anti CD20	0.024–0.196
		Mosunetuzumab	11,618.9	11,617.9 ± 0.4	IgGK	Anti CD20	17.9
			12,068.0	12,066.6 ± 0.5	IgGK	Anti CD3	

* The determination of belantamab was performed in duplicate due to limited drug availability. EXENT^®^ detectability threshold: 0.0015 g/dL (15 µg/mL). 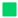 Detectable: t-mAbs with Cmax ≥ 15 µg/mL. 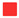 Not detectable: t-mAbs with Cmax < 15 µg/mL. MM: multiple myeloma; Lymph: lymphomas; SLAMF7: Signaling Lymphocytic Activation Molecule Family member 7; BCMA: B-cell maturation antigen; SC: subcutaneous; IV: intravenous.

## Data Availability

The data presented in this study are available within the article. Further information can be obtained from the corresponding author upon reasonable request.
